# Occurrence of *Campylobacter jejuni* in Gulls Feeding on Zagreb Rubbish Tip, Croatia; Their Diversity and Antimicrobial Susceptibility in Perspective with Human and Broiler Isolates

**DOI:** 10.3390/pathogens9090695

**Published:** 2020-08-24

**Authors:** Luka Jurinović, Sanja Duvnjak, Gordan Kompes, Silvija Šoprek, Borka Šimpraga, Fani Krstulović, Marina Mikulić, Andrea Humski

**Affiliations:** 1 Croatian Veterinary Institute, Poultry Centre, Laboratory for Bacteriology, 10000 Zagreb, Croatia; b_simpraga@veinst.hr (B.Š.); f_krstulovic@veinst.hr (F.K.); 2 Croatian Veterinary Institute, Department for Bacteriology and Parasitology, Laboratory for Bacterial Zoonoses and Molecular Diagnostics of Bacterial Diseases, 10000 Zagreb, Croatia; marjanovic@veinst.hr; 3 Croatian Veterinary Institute, Department for Bacteriology and Parasitology, Laboratory for General Bacteriology and Mycology, 10000 Zagreb, Croatia; kompes@veinst.hr; 4 Department of Clinical Microbiology, University Hospital for Infectious Diseases, 10000 Zagreb, Croatia; silvija.soprek@gmail.com; 5 Croatian Veterinary Institute, Department for Veterinary Public Health, Laboratory for Food Microbiology, 10000 Zagreb, Croatia; mikulic@veinst.hr (M.M.); humski@veinst.hr (A.H.)

**Keywords:** MLST, wild birds, gulls, *Laridae*, AMR

## Abstract

Campylobacteriosis is the most common gastrointestinal bacterial disease in the European Union (EU). Wild birds are one of the natural reservoirs of these pathogens. In this study we tested cloacal swabs of 643 gulls captured on rubbish tip in Zagreb, Croatia for the presence of *Campylobacter* spp. and found 168 *Campylobacter* positive samples. We used multilocus sequence typing (MLST) to genotype 62 random *C. jejuni* isolates from gulls, 24 isolates from broiler caeca, 27 isolates from broiler neck skins and 23 human isolates. Altogether, we identified 44 different STs, from which 19 were newly described. Most of the new STs (14) originate from gulls. Although humans and broilers share the majority of STs and isolates from gulls are separated from these, there was one ST present in all three hosts: 45. Additionally antimicrobial susceptibility to six antimicrobials was performed on 123 *C. jejuni* strains isolated from broiler caeca (*n* = 22), neck skins of broilers (*n* = 20), gulls cloacal swabs (*n* = 50) and human faeces (*n* = 31) by the broth microdilution method. Results show lower resistance of gull isolates to NAL and CIP, while resistance to TET was as high as in human and broiler isolates.

## 1. Introduction

*Campylobacter* is the most commonly reported gastrointestinal bacterial pathogen in humans in the European Union (EU), and it has been so since 2005 [1].

A variety of animals constitute a natural reservoir for these bacteria, including domestic animals: cats, dogs, cows, pigs, poultry, and wild birds, with the avian reservoir being the most important [2]. As studies documenting *C. jejuni* prevalence in wild animals have increased rapidly in the past 15 years [3,4,5,6], many of them showed that diverse groups of wild birds harbour this species [7]. In the last decades, there has been an increase in the incidence of campylobacteriosis worldwide [1,2,8,9]. This zoonosis is therefore of great economic and public concern [10].

The emergence of antimicrobial resistance among *Campylobacter* isolates (of both human and animal origin) has increased dramatically in all groups of antimicrobials, particularly focusing on high resistance rates to the group of fluoroquinolones, which have rapidly increased in the past decade. Monitoring the progress of all of the stated facts indicates we are dealing with a severe public health issue due to antimicrobial resistance that in recent years has become a major public health concern in countries regardless to their national income [11,12].

Gulls are one of the most common birds in human surroundings. They are present in most aquatic habitats and from sea coasts and ports to lakes and rivers. In recent decades, wherever and whenever there was no separation of organic component of household garbage, they found an endless source of food on open rubbish tips [13,14]. Many of them gather on rubbish tips near bigger towns. During our previous research, we got an insight into their movements trough different ringing programs [15,16] and gulls that feed in winter on Zagreb city rubbish tip origin from all over Europe. As gulls are capable of crossing great distances in short periods, and tend to form huge flocks of individuals of different species from different populations and age groups on adequate feeding sites; they can easily transmit and spread pathogens on huge areas and therefore can potentially pose a serious threat to both humans and farm animals.

In this paper we present the molecular study of *C. jejuni* isolates from gulls, poultry and humans using multilocus sequence typing (MLST), and differences in antimicrobial resistance between isolates from different hosts found in Croatia.

## 2. Results

### 2.1. MLST

A total of 643 gulls of five species (Yellow-legged Gull, *Larus michahellis* (*n* = 235); Black-headed Gull, *L. ridibundus* (*n* = 376); Caspian Gull, *L. cachinanns* (*n* = 14), Herring Gull, *L. argentatus* (*n* = 2) and Common Gull, *L. canus* (*n* = 16)) were captured during winter months (November–March) in the period between November 2016 and March 2020 on Zagreb city rubbish tip. Cloacal swabs were taken from all the birds in order to study occurrence of *C. jejuni*.

There were 168 swabs positive for the presence of *Campylobacter* spp. (26.1%). Highest isolation rate was from Black-headed Gulls (118 or 31.4%), followed by Caspian (3 or 21.4%), Common (3 or 18.8%) and Yellow-legged Gulls (44 or 18.7%). The most prevalent *Campylobacter* species was *C. jejuni* (148 or 88.1%) followed by *C. lari* (19 or 11.3%) and *C. coli* (1 or 0.6%). 

MLST was performed on 136 *C. jejuni* isolates originating from gulls, broiler caeca, broiler neck skins and human feces. All isolates gave full sequence type (ST) profiles. In this study 19 new ST profiles were identified as follows: 14 from gulls (9446, 9447, 9458, 9845, 9846, 9847, 9850, 9851, 9852, 9853, 9854, 9855, 9856 and 10,297), three from broiler neck skins (10,230, 10,231 and 10,232) and two from broiler caeca (10,228 and 10,229). In addition, four new alleles were described: two *pgm* from gulls (1007 and 1008), one *glyA* from broiler caeca (832) and one *glnA* from broiler neck skins (747) (Table 1).

In total, we identified 44 different sequence types (STs) belonging to 18 clonal complexes (CCs) and 15 singletones (STs not belonging to any CCs). Gulls have shown greater diversity in number of STs and isolates from broiler neck skins in number of CCs (Table 1).

The most frequent CCs were ST-1275 (found exclusively in gulls) and ST-21 (found in other three hosts). 

### 2.2. AMR

Distribution of MIC (minimum inhibitory concentration) values among *C. jejuni* strains isolated from broiler caecum, neck skins of broilers, gulls’ cloacal swabs and human faeces are shown in Table 2, Table 3, Table 4 and Table 5, while MIC_50_/MIC_90_ values are shown in Table 6.

According to antibiotic susceptibility testing by the broth microdilution method, all *C. jejuni* strains isolated from broiler caecum, broiler neck skins and human faeces were found to be susceptible to ERY and GEN. *C. jejuni* strains isolated from gull cloacal swabs were also found to be susceptible to ERY, while 2.0% of isolates from gull cloacal swabs were found to be resistant to GEN. Very high resistance of *C. jejuni* isolates from broiler caecum and broiler neck skins to CIP and NAL (90.9% and 90.0%) was found. High resistance to CIP and NAL was also observed in human *C. jejuni* isolates (74.2% and 80.6%). A high proportion of isolates were resistant to TET (40.9% broiler caecum, 45.0% broiler neck skins, 46.0% gull cloacal swabs and 38.7% human faeces). Resistance to STR of *C. jejuni* isolates from broiler caecum, broiler neck skins, human faeces and gull cloacal swabs was 4.5%, 5.0%, 9.7% and 8.0%, respectively.

The lowest MIC_50_ values (≤0.5–2 mg/L) were obtained for the ERY, TET, GEN and STR while low MIC_90_ values (≤1–0.5 mg/L) were observed for ERY and GEN (with the exception of isolates from gull cloacal swabs; ERY = 2 mg/L).

The highest MIC_90_ values were observed for TET, CIP and NAL.

## 3. Discussion

Although the overall prevalence of *C. jejuni* in our study is a bit smaller than the mean one found in literature [7], seasonality should be taken in consideration. Broman et al. [17] found that the prevalence of *C. jejuni* in Black-headed Gulls is much higher in autumn (37.6–59.6%) than in winter (7.0–21.6%), and our results are in the higher end of their winter values. 

ST-1275 was the only CC found in all gull species (except for Herring Gull, where we did not isolate any *Campylobacter* spp., probably due to the small number of examined samples (two birds)), with 39 isolates from 16 STs. It was not found in broiler or human isolates. This CC is usually associated with wild birds mainly from family *Laridae* [18,19]. This is in line with our findings, as STs from ST-1275 CC were the same ones found in other gull species, like Black-headed Gulls from Sweden (1223, 1268, 1275, 1341), Silver Gulls from Australia (3049), the American Herring Gull from the USA (1268), the Ring-billed Gull from the USA (637, 1275), gull (species not written) from Canada (1275) or seagull (species not written) from Spain (9239). The second largest group was singletons (*n* = 15) (STs not matching any CCs). Most of the singletons were found on gulls exclusively, while only two were found in broiler samples. Those found in gull samples during this study are usually also found on gulls (2351 and 2654), other wild birds (2654, 9209, 9220 and 9221) or environmental waters (5053). Unfortunately, in PubMLST it is not always listed the exact species or even genus of wild bird from which the strains are isolated. This data would give us a better picture about species preferences for each ST especially as they are linked to gulls from all over the world, from Australia, New Zealand, China, USA, Canada, Sweden, etc. Similar case is described in Blackbirds from Sweden, Australia and Azores that share same CCs although separated for hundreds of generations and not connected with migration routes [20].

**Figure 1:**
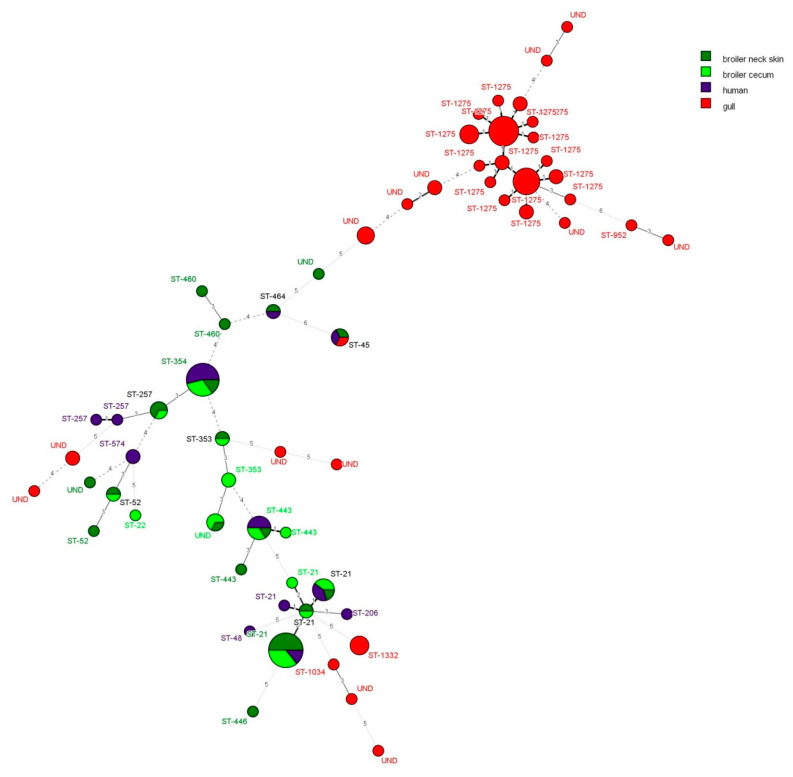
Minimum spanning tree of *C. jejuni* isolates. Isolates are labeled according to the clonal complex they belong to. Differences between samples are expressed as the number of allele differences between them (number of locus variants). In addition, a thicker solid line corresponds to 1 locus variants; a thicker dotted line corresponds to 2 locus variants; thinner solid line corresponds to 3 locus variants; a thinner dashed line corresponds to 4 locus variant and a thinner dotted line corresponds to 5 locus variants.

In humans most common CCs were ST-354 (7 isolates of 1 ST) and ST-21 (5 isolates of 3 STs). In broilers, there is an almost complete overlap between isolates from caeca and neck skins, so we analysed those two groups as a single. The most common CCs were ST-21 (18 isolates from 4 STs) and ST-354 (6 isolate of 1 ST). It is interesting that none of these two CCs were identified in smaller scale MLST study done on chicken meat in Croatia by Mikulić et al. [21]. As suggested in previous studies, humans and chicken share a great part of *C. jejuni* STs and CCs (Figure 1.). This relationship is visible in minimum spanning tree (Figure 1) where most of the broiler and human isolates are close together sharing same CCs and are distant from gull’s isolates. Out of 14 different CCs found in broilers and humans, six were found in both hosts, while 5 and 3 were found in broilers and humans alone, respectively.

In this study we found only one ST (also only one CC) shared by all three host types and it is 45 (ST-45). Together with ST-21, ST-45 is in the group of most common STs found between human isolates [22,23,24]. It is known that ST 45 can be found in many species of wild birds, like passerines, pigeons, geese, owls, rails etc. [18,20,25]. As gulls sampling for this study was done on rubbish tip where gulls find endless source of food in human leftovers, we believe that this infection started from humans and/or chicken, rather than the other way around, but there is more research to be done (especially whole genome sequencing) to get better insight.

In the present study, we investigated the in vitro susceptibility of 123 *C. jejuni* strains isolated from broiler caeca (*n* = 22), neck skins of broilers (*n* = 20), gulls’ cloacal swabs (*n* = 50) and human faeces (*n* = 31) by the broth microdilution method to six antimicrobials.

Results obtained in our study showed very high resistance of *C. jejuni* isolates from broilers and humans to fluoroquinolones (74.2–90.9%) and tetracycline (38.7–45.0%). High resistance rates to CIP, NAL and TET in broilers and humans was reported from the majority of EU countries [26]. As previously established, an overuse of antimicrobial agents in the poultry, which is the main reservoir of *Campylobacter* spp., takes responsibility for the alarming rate of AMR to fluoroquinolones [27]. In JIACRA report [28], it was stated that there is statistically-significant positive associations between the consumption of fluoroquinolones and tetracyclines in food-producing animals and resistance to fluoroquinolones and tetracyclines in *C. jejuni* from such animals. Furthermore, it is mentioned that statistically-significant associations were observed between the occurrence of resistance to fluoroquinolones and tetracyclines in *C. jejuni* from food-producing animals and the occurrence of resistance in *C. jejuni* from human infections. Those observations, regarding fluoroquinolones resistance, could be supported by the fact that we found moderate resistance to CIP and NAL in *C. jejuni* isolates from gull (34.0% and 32.0%), i.e., in wild birds that have not been given or treated with antimicrobials. Quite opposite, high resistance to TET in *C. jejuni* isolates from gull (46.0%) cannot be explained by this report, nor with the results reported earlier in studies of *C. jejuni* isolated from gulls and wild birds [19,29,30,31].

In present study, resistance to erythromycin and gentamicin was not detected for *C. jejuni* isolated from broilers and humans. In addition, low resistance rate in broiler and human isolates was found to streptomycin (4.5–9.7%). These results are similar to those published earlier [26,32]. 

Regarding *C. jejuni* isolates from gull, all isolates were found to be susceptible to erythromycin, while only one isolate (2.0%) was found to be resistant to gentamicin. Four isolates (8.0%) were resistant to streptomycin. This is concordance with previous publications [19,25,31].

## 4. Materials and Methods

In the period between November 2016 and March 2020 gulls were captured using cannon net on Zagreb city rubbish tip (45.765 N 16.025 E) in order to collect cloacal swabs. 

Taken swab samples were tested for the presence of thermophilic *Campylobacter* spp. according to the EN ISO 10272-1 method. 

Isolates from broilers (*Gallus gallus* chicks bred for meat production) were collected at the Laboratories of the Croatian Veterinary Institute during the implementation of national monitoring programs for *Campylobacter* in broilers(according to amendment of Regulation (EC) 2073/2005 to include *Campylobacter* process hygiene criterion [33]; Regulation (EC) No 2160/2003 [34]), while human isolates were collected in the Infectious Diseases Clinic “Dr. Fran Mihaljević” from gastroenteritis patients, all during 2019 and 2020. 

Bacterial DNA was extracted from fresh bacterial culture by boiling for 20 min at 95 °C and centrifuging at 14,000× *g* for 60 s. Species determination was performed using multiplex PCR [35]. Multilocus sequence typing (MLST) was performed on 62 randomly chosen *C. jejuni* isolates from gulls. In addition, MLST was performed on 24 isolates from broiler caeca, 27 isolates from broiler neck skins and 23 human isolates. PCR products were sequenced at Macrogen Europe (The Netherlands).

Sequences were edited using BioEdit software. Sequence types (ST) and clonal complexes (CC) were determined using *Campylobacter* multilocus sequence typing website (https://pubmlst.org/campylobacter/) sited at the University of Oxford [36].

Minimum spanning tree was done using BioNumerics 7.6.3 version (BioMerieux, Applied Maths, Sint-Martens-Latem, Belgium). We used advanced cluster analysis. The maximum number of n-locus variants was 5, where *n* = 1 locus variants have the highest weight (10,000), *n* = 2 have 1 000 times smaller (10) and all others 10 times smaller (1).

Antimicrobial resistance of 123 *C. jejuni* isolates isolated from broiler caecum (*n* = 22), neck skins of broilers (*n* = 20), gulls’ cloacal swabs (*n* = 50) and human faeces (*n* = 31) was tested. The antimicrobial susceptibility testing was determined by the broth microdilution method for six antimicrobials, following EUCAST (European Committee on Antimicrobial Susceptibility Testing) guidelines [37]. EUCAMP2 microplate (Sensititer, Trek Diagnostic Systems Ltd. East Grinstead, West Sussex, RH19 1XZ, UK) was used for the susceptibility testing of erythromycin (ERY; 1–128 mg/L), ciprofloxacin (CIP; 0.12–16 mg/L), tetracycline (TET; 0.5–64 mg/L), gentamicin (GEN; 0.12–16 mg/L), nalidixic acid (NAL; 1–64 mg/L) and streptomycin (STR; 0.25–16 mg/L). Before susceptibility testing, isolates were revived on blood agar supplemented with 5% of defibrinated sheep blood (blood agar base no. 2, Merck; 5% DSB, BIOGNOST, Zagreb) and incubated in microaerobic atmosphere (CampyGen, Thermo Scientific) at 42 °C for 24 h. 

EUCASTepidemiological cut-off values were used for interpretative thresholds for resistance. 

MIC50 and MIC90 levels were defined as the lowest concentration of the antibiotic at which 50% and 90% of the isolates were inhibited, respectively. 

Reference strains *C. jejuni* ATCC 33,560 was used to ensure that the results were within acceptable limits of quality control for susceptibility testing.

## Figures and Tables

**Table 1:** List of sequence types (ST) and clonal complexes (CC) found in three different host types (gulls, broilers and humans) in Croatia. New STs and alleles are printed **bold.**

CC	ST	aspA	glnA	gltA	glyA	pgm	tkt	uncA	*Larus ridibundus*	*Larus michahellis*	*Larus cachinnans*	*Larus canus*	Broiler Caeca	Broiler Neck Skins	Human	∑
st-1275	637	10	33	22	49	43	82	31	7	2	1					10
	1223	27	33	22	49	43	9	31	6	1	1					8
	1268	27	33	22	49	43	20	31	1							1
	1275	27	33	22	49	43	82	31	4							4
	1341	10	33	22	49	43	9	31	2							2
	3049	27	33	95	49	43	9	31	2							2
	3629	228	33	22	49	43	82	31		1						1
	4119	10	33	22	6	43	82	31	2							2
	9239	27	33	22	49	925	9	31	1							1
	**9446**	10	33	22	49	101	9	31		1						1
	**9447**	10	33	22	97	43	9	31		1						1
	**9846**	27	33	22	49	116	10	47		1						1
	**9847**	10	33	22	49	115	82	31			1					1
	**9852**	10	31	22	49	43	82	31				1				1
	**9855**	27	33	22	49	**1007**	9	31	2							2
	**9856**	10	33	22	49	**1008**	82	31		1						1
st-1332	1332	2	1	29	28	58	25	58	4							4
st-1034	**10,297**	2	15	4	49	2	25	23	1							1
st-952	**9850**	64	22	78	98	116	86	16	1							1
ST-574	305	9	53	2	10	11	3	3							2	2
ST-464	464	24	2	2	2	10	3	1						1	1	2
ST-460	460	24	30	2	2	89	59	6						1		1
	**10,230**	24	24	420	2	11	59	6						1		1
ST-446	**10,232**	47	**747**	5	10	11	61	8						1		1
ST-443	51	7	17	2	15	23	3	12					2	1	3	6
	**10,229**	7	17	2	15	26	3	12					1			1
	**10,231**	79	17	2	337	23	2	12						1		1
ST-354	354	8	10	2	2	11	12	6					4	2	7	13
ST-353	356	14	17	5	2	11	3	6					1	1		2
	2036	7	17	52	10	11	3	6					2			2
ST-257	257	9	2	4	62	4	5	6							1	1
	367	2	2	4	62	4	5	6							1	1
	824	9	2	2	2	11	5	6					1	2		3
ST-206	572	62	4	5	2	2	1	5							1	1
ST-52	2066	9	10	5	10	22	3	6						1		1
	2100	9	25	2	10	22	3	8					1	1		2
ST-48	475	2	4	1	4	19	62	5							1	1
ST-45	45	4	7	10	4	1	7	1	1					1	1	3
ST-22	**10,228**	1	3	6	**832**	3	3	3					1			1
ST-21	19	2	1	5	3	2	1	5					1	1		2
	50	2	1	12	3	2	1	5					2	1	2	5
	822	2	1	79	3	2	1	5						1	1	2
	883	2	17	2	3	2	1	5					1			1
	6175	2	1	5	10	608	1	5					5	7	2	14
UND	881	9	17	52	10	10	3	1					2	1		3
	1080	10	2	107	137	120	76	1						1		1
	1970	37	29	75	48	126	5	23	1							1
	2133	55	21	2	71	11	37	3						1		1
	2351	10	31	63	129	101	45	49	1							1
	2654	10	31	95	268	101	134	1	2	1						3
	5053	1	2	95	62	472	400	147	2							2
	9209	10	31	63	384	101	9	49	2							2
	9220	487	280	5	47	57	20	6	1							1
	9221	178	6	34	47	394	722	6	1							1
	**9458**	2	15	95	48	13	25	23	1							1
	**9845**	7	172	21	49	936	9	31		1						1
	**9851**	10	8	34	6	39	7	31	1							1
	**9853**	1	172	269	62	116	377	147	1							1
	**9854**	10	8	22	6	39	20	3	1							1
									48	10	3	1	24	27	23	136

**Table 2:** Distribution of MIC values among *C. jejuni isolates* from broiler caecum.

	ERY	CIP	TET	GEN	NAL	STR
128						
>64			6		19	
64			3		1	
32						
>16		3				
16		9				
8		8			1	1
4					1	3
2						5
1			1	3		10
≤1	22					
>0.5						
0.5				9		3
≤0.5			12			
0.25				9		
0.12						
≤0.12		2		1		

**Table 3:** Distribution of MIC values among *C. jejuni isolates* from neck skins of broilers.

	ERY	CIP	TET	GEN	NAL	STR
128						
>64			4		11	
64			2		7	
32			3			
>16						
16		5				
8		13				1
4					2	
2	1					
1						12
≤1	19					
>0.5						
0.5				4		7
≤0.5			11			
0.25				9		
0.12						
≤0.12		2		7		

**Table 4:** Distribution of MIC values among *C. jejuni isolates* from gull cloacal swabs.

	ERY	CIP	TET	GEN	NAL	STR
128						
>64			8		16	
64			8			
32			2			
>16						1
16			3		1	2
8		1	1		15	1
4	2		1	1	18	5
2	10	16				20
1			1	1		21
≤1	38					
>0.5						
0.5		1		37		
≤0.5			26			
0.25		5		11		
0.12						
≤0.12		27				

**Table 5:** Distribution of MIC values among *C. jejuni isolates* from human faeces.

	ERY	CIP	TET	GEN	NAL	STR
128						
>64			8		15	
64			2		8	
32			2		2	
>16						1
16		10				2
8		13			3	
4					3	
2						6
1			1	1		17
≤1	31					
>0.5						
0.5				12		5
≤0.5			18			
0.25		1		9		
0.12						
≤0.12		7		9		

**Table 6:** MIC_50_ and MIC_90_ values.

		MIC_50_ (mg/L)	MIC_90_ (mg/L)	S (N/%)	R (N/%)	EUCAST Epidemiological Cut-Off Value
						R
ERY	BC	≤1	≤1	22/100	0/0	>4
	BNS	≤1	≤1	20/100	0/0	
	GS	≤1	2	50/100	0/0	
	HF	≤1	≤1	31/100	0/0	
CIP	BC	16	>16	2/9.1	20/90.9	>0.5
	BNS	8	16	2/10.0	18/90.0	
	GS	≤0.12	2	33/66.0	17/34.0	
	HF	8	16	8/25.8	23/74.2	
TET	BC	≤0.5	>64	13/59.1	9/40.9	>1
	BNS	≤0.5	>64	11/55.0	9/45.0	
	GS	≤0.5	>64	27/54.0	23/46.0	
	HF	≤0.5	>64	19/61.3	12/38.7	
GEN	BC	0.5	1	22/100	0/0	>2
	BNS	0.25	0.5	20/100	0/0	
	GS	0.5	0.5	49/98.0	1/2.0	
	HF	0.5	0.5	31/100	0/0	
NAL	BC	>64	>64	2/9.1	20/90.9	>16
	BNS	>64	>64	2/10,0	18/90.0	
	GS	8	>64	34/68.0	16/32.0	
	HF	64	>64	6/19.4	25/80.6	
STR	BC	1	4	21/95.5	1/4.5	>4
	BNS	1	1	19/95.0	1/5.0	
	GS	2	4	46/92.0	4/8.0	
	HF	2	16	28/90.3	3/9.7	

BC—broiler caecum; BNS—broiler neck skins; GS—gull cloacal swab; HF—human faeces.
